# Exploring the Discrepancies in Actual and Perceived Benefits of Dietary Supplements Among Obese Patients

**DOI:** 10.3233/SHTI190491

**Published:** 2019-08-21

**Authors:** Zhe He, Laura A. Barrett, Rubina Rizvi, Seyedeh Neelufar Payrovnaziri, Rui Zhang

**Affiliations:** a School of Information, Florida State University, Tallahassee, Florida, USA,; b Institute for Health Informatics and Department of Pharmaceutical Care & Health Systems, University of Minnesota, Minneapolis, Minnesota, USA

**Keywords:** Obesity, Dietary Supplements, Nutrition Surveys

## Abstract

Dietary supplements (DSs) have gained increased popularity for weight loss due to its availability without prescription, relatively low price, and ease of use. Consumers with limited health literacy may not adequately know the benefits and risks associated with DSs. In this project, we found a knowledge gap between reported benefits of major DSs by adults with obesity in the National Health and Nutrition Examination Survey 2003–2014 and those reported in existing DS knowledge databases.

## Introduction

Obesity, a complex chronic medical condition having multifactorial etiology, is on the rise not only in the US but also around the globe [1]. In the US, the prevalence of obesity among the 18 years or older population has increased from 33.7% in 2007–2008 to 39.6% in 2015–2016; whereas, the prevalence of severe obesity in adults has increased from 5.7% in 2007–2008 to 7.7% in 2015–2016 [2]. Many patients who are overweight and obese consider using dietary supplements (DSs) for weight loss. Currently, there are a number of available options out there, either to simply maintain a healthy weight or to actually treat people who are overweight/obese and are at high risk of weight-related comorbidities; however, the vast majority of treatment around lifestyle changes often fail due to noncompliance resulting from various factors e.g., poor motivation, lack of time, gaps in knowledge/awareness, and lack of strong and prolonged commitment to observe the actual results [3]. On the other hand, most of the pharmacological and surgical procedures are associated with a substantial health risk at a high cost [4].

The popularity of complementary alternative medicine, especially the use of dietary supplements for weight management, has gained much popularity. Aside from the health benefits resulting from weight loss, there are various other reasons (several of them being misconceptions) for people to turn to DSs for losing weight and/or maintaining a healthy weight, such as DSs being a natural product, effective, fast acting, associcated with minimal side effects, easily available, and at relatively low price. Often consumers swtich to DSs in frustration resulting from failures in previous weight loss attempts following strict diet and exercise regimens.

Interestingly. earlier studies have revealed that use of DSs is more commonly preventive with an aim to maintain and improve overall health, rather than being therapeutic to treat obesity. In this project, we aim to gain a better understanding of the use of DS products among obese people as well as their perceived benefits of these products. We used the combined National Health and Nutrition Examination Survey (NHANES) data from 2003–2014 to answer two research questions (RQ): RQ1: What are the perceived benefits of DS usage among obese patients? RQ2: Is there a knowledge gap between their perceived benefits and those reported in existing DS knowledge bases?

## Methods

NHANES is a continuous cross-sectional health survey conducted by the National Center for Health Statistics [5]. It evaluates a stratified multistage probability sample of the non-institutionalized US population. We first extracted the demographic, examination, and dietary data from NHANES for survey years 2003 – 2014 (6 survey cycles). To strengthen the analytical power of the study, survey data from multiple survey cycles were combined for the following analyses. Inclusion criteria for the cohort include: (1) BMI ≥ 30 kg/m^2^, and (2) age ≥ 18. This left us with a cohort of 11,959 participants. DS use was pulled for this cohort. Total and individual DS use was available for all survey cycles although detailed data was inconsistent for years 2003–2004 and 2007–2008. These inconsistencies caused minor issues with data processing but not with the data validity. This data was used for the demographic data. DSs used for specific reasons were grouped into types based on product information for the analysis.

### Data Analysis

We first created a profile of obese adults in the cohort with respect to gender, age, race, and household income. Using the 2007–2014 data with the needed variables, we assessed the major perceived benefits of the DSs used by obese adults in the cohort, stratified by specific DS type. In our previous study [6], we learned that the Natural Medicine Comprehensive Database (NMCD) [7] is the most comprehensive resource, providing DS information that is reliable, clinically relevant, and evidence-based. Hence, in this study, we compared the reported benefits of obese adults in NHANES with NMCD aiming to identify the knowledge gap.

## Results

Out of 11,959 survey participants, 5,421 (45.3%) self-reported taking at least one DS. The maximum number of DS used by an individual was 20. Survey participants were taking a total of 8,057 DSs , out of which they took 5,591 (69.4%) DSs on their personal will, and they took the remaining 2,466 (30.6%) DSs on their clinicians’ advice. We looked at individual reasons for DS use between the years 2007–2014. Participants were given a list of top five reasons for DSs intake where they could choose 1 or more options (Table 1). We also looked at DS use reasons ‘*For Weight Loss*’ and ‘*To Maintain Blood Sugar/Diabetes*’. These reasons were matched to specific DS type. Overall, this same group (2007–2014) used 6,929 DSs.

In addition, we investigated if the information provided in the existing knowledge base aligns with the reported use of a particular DS. We found consistency between the reported use of a particular DS and its use/effectiveness as provided in the existing knowledge base (NMCD) for improving certain conditions, e.g. general overall health, bone/joint health, heart health/cholesterol, and as a dietary supplement. In fact, we found useful information about the primary use of a DS in addition to its other common uses, e.g. use of calcium for bone and joint health other than to improve general health. For the remaining three conditions, i.e., getting energy, losing weight, and maintaining blood sugar, we found that consumers were taking DSs indiscriminately without sufficient, current, and scientific knowledge on how a particular DS impacts the human body, e.g. the use of MVMM and Vitamin B-complexes for weight loss and/or maintaining blood sugars among diabetic patients, rather than the intended role of a diet supplement in people with restricted diets.

## Discussion and Conclusions

In this study, we used the NHANES data to assess the use and perceived benefits of dietary supplements among obese adults. Demographics clearly play a role in DS use. We found that females were more likely than males to use DSs. With respect to age, the older the respondent was, the more likely he or she used at least one DS. These results conclude that there is lack of adequate knowledge about specific DS use and their resulting benefits, esepcially among consumers who are taking DSs for obesity and/or related conditions. Even though they consider obesity as a health concern, they are not using DSs for actual weight loss. This includes the use of MVMM and Vitamin B complexes for losing weight and/or maintaining blood sugar among diabetic patients rather than their actual dietary supplment role for people with restricted diets. We found that consumers were taking DSs indiscriminately without sufficient, current, and scientific knowledge on how a particular DS actually impacts the human body e.g., use of calcium for heart health, despite a considerable number of risks [8]. Overall, we found NMCD to be reasonable in finding the relevant information for most ingredients/products despite few challenges. This study pinpoints the gaps of the perceptions of dietary supplements among the general public, thereby informing informaticians of the opportunities for developing informatics tools to disseminate the “accurate and factual” knowledge to the consumers.

## Figures and Tables

**Table 1–:** Reason for DS use matched to type of DS

Reason (Total Responses)	Highest Type	# of responses for this DS type	% of total responses for this reason
General Overall Health^a^ (5,313)	MVMM^c^	1,677	41.0%
Bone and Joint Health^b^ (1,569)	Calcium / Bone/ Joint	834	57.7%
To Supplement Diet/Food Not Enough (941)	MVMM	462	49.1%
Heart Health/Cholesterol (787)	Omega-3	410	52.1%
To Get More Energy (737)	MVMM	316	42.9%
For Weight Loss (184)	Botanical^d^	55	29.9%
To Maintain Blood Sugar/Diabetes (125)	Botanical^d^	40	32.0%

a: Includes: To prevent health problems (813), to improve my overall health (2170), to maintain health/to stay healthy (1734), and to prevent colds/boost immune system (596). 4090 total responses once duplicative response were removed.

b: Includes: For healthy joints/arthritis and for bone (522) health/build strong bones/osteoporosis (1047). 1446 total responses once duplicative responses were removed.

c: Multivitamins/multiminerals

d: DS classified as a botanical if it is part of a plant, tree, shrub, herb, etc.
